# *KRAS* mutation leads to decreased expression of regulator of calcineurin 2, resulting in tumor proliferation in colorectal cancer

**DOI:** 10.1038/oncsis.2016.47

**Published:** 2016-08-15

**Authors:** H Niitsu, T Hinoi, Y Kawaguchi, K Sentani, R Yuge, Y Kitadai, Y Sotomaru, T Adachi, Y Saito, M Miguchi, M Kochi, H Sada, M Shimomura, N Oue, W Yasui, H Ohdan

**Affiliations:** 1 Department of Gastroenterological and Transplant Surgery, Applied Life Sciences, Institute of Biomedical & Health Sciences, Hiroshima University, Hiroshima, Japan; 2 Department of Surgery, Institute for Clinical Research, National Hospital Organization Kure Medical Center and Chu-goku Cancer Center, Kure City, Hiroshima, Japan; 3 Department of Surgery, Tsuchiya General Hospital, Hiroshima, Japan; 4 Department of Molecular Pathology, Institute of Biomedical & Health Sciences, Hiroshima University, Hiroshima, Japan; 5 Department of Gastroenterology and Metabolism, Applied Life Sciences, Institute of Biomedical & Health Sciences, Hiroshima University, Hiroshima, Japan; 6 Natural Science Center for Basic Research and Development, Hiroshima University, Hiroshima, Japan; 7 Department of Surgery, Hiroshima General Hospital of West Japan Railway Company, Hiroshima, Japan; 8 Department of Surgery, Hiroshima City Funairi Citizens Hospital, Hiroshima, Japan; 9 Department of Surgery, National Hospital Organization Higashihiroshima Medical Center, Hiroshima, Japan

## Abstract

*KRAS* mutations occur in 30–40% of all cases of human colorectal cancer (CRC). However, to date, specific therapeutic agents against *KRAS*-mutated CRC have not been developed. We previously described the generation of mouse models of colon cancer with and without *Kras* mutations (*CDX2P-G22Cre;Apc*^*flox/flox*^*; LSL-Kras*^*G12D*^ and *CDX2P-G22Cre;Apc*^*flox/flox*^ mice, respectively). Here, the two mouse models were compared to identify candidate genes, which may represent novel therapeutic targets or predictive biomarkers. Differentially expressed genes in tumors from the two mouse models were identified using microarray analysis, and their expression was compared by quantitative reverse transcription–PCR (qRT–PCR) and immunohistochemical analyses in mouse tumors and surgical specimens of human CRC, with or without *KRAS* mutations, respectively. Furthermore, the functions of candidate genes were studied using human CRC cell lines. Microarray analysis of 34 000 transcripts resulted in the identification of 19 candidate genes. qRT–PCR analysis data showed that four of these candidate genes (*Clps*, *Irx5*, *Bex1* and *Rcan2*) exhibited decreased expression in the *Kras*-mutated mouse model. The expression of the regulator of calcineurin 2 (*RCAN2)* was also observed to be lower in *KRAS*-mutated human CRC. Moreover, inhibitory function for cancer cell proliferation dependent on calcineurin was indicated with overexpression and short hairpin RNA knockdown of *RCAN2* in human CRC cell lines. *KRAS* mutations in CRC lead to a decrease in *RCAN2* expression, resulting in tumor proliferation due to derepression of calcineurin–nuclear factor of activated T cells (NFAT) signaling. Our findings suggest that calcineurin–NFAT signal may represent a novel molecular target for the treatment of *KRAS*-mutated CRC.

## Introduction

It is widely known that mutations in the Kirsten rat sarcoma viral oncogene homolog *(KRAS)* gene occur in the early stages of the adenoma–adenocarcinoma sequence in human colorectal cancer (CRC) development.^1^ *KRAS*, along with the neuroblastoma rat sarcoma viral oncogene homolog *(NRAS)* and Harvey rat sarcoma viral oncogene homolog *(HRAS)* genes, is a member of the *RAS* gene family. *KRAS* and *NRAS,* which have been shown to exhibit somatic mutations in 30–40% and 2–5% of human CRCs, respectively,^2, 3, 4^ are known predictors for resistance to treatment with anti-epidermal growth factor receptor antibodies.^5, 6, 7, 8^ Although these biomarkers have enabled the development of individualized therapies, especially for the treatment of CRCs with wild-type *RAS*, specific therapeutic agents against *RAS*-mutated CRC have not yet been established. *RAS* mutations cause oncogenic activation independent of epidermal growth factor–epidermal growth factor receptor signal transmission, resulting in a lack of response to anti-epidermal growth factor receptor antibodies.^9^ To overcome this resistance mechanism, the identification of downstream molecules that show potential as new therapeutic targets for *RAS-*mutated CRC has become an important focus.

Calcineurin, which was first cloned in neural cells as a Ca^2+^- and calmodulin-dependent serine/threonine protein phosphatase,^10, 11, 12^ dephosphorylates nuclear factor of activated T cells (NFAT). NFAT subsequently translocates into the nucleus, where it acts as a transcription factor that promotes various cellular activities.^13, 14, 15^ Calcineurin is widely distributed and its role has been characterized in various organs, including the myocardium,^15, 16, 17^ skeletal muscle^16^ and lymphocytes.^15, 18^ Its immunological function has been studied extensively, and calcineurin inhibitors (CNIs), such as cyclosporine A and tacrolimus, are widely used for immunosuppression.^19, 20, 21^ However, little is known about the contribution of calcineurin to CRC, although previous reports have indicated the oncogenic function of calcineurin: cycolooxgenase-2 and prostaglandin E2 are induced by the pharmacological stimulation of the Ca^2+^–calcineurin–NFAT signaling pathway in CRC cell lines,^22^ and the expression of calcineurin A specifically increases in human CRCs, resulting in the activation of phosphatase activity of calcineurin.^23, 24^

We have previously developed mouse models in which sporadic colon cancers were generated via the colonic epithelium-specific inactivation of adenomatous polyposis coli (*Apc*), with and without the activation of *Kras,* using the *loxP/Cre* system.^25, 26, 27, 28^ Gene expression profiling of the two mouse models revealed that *Glut-1*, which is upregulated in *KRAS*-mutated human CRC cell lines,^29^ is also upregulated in tumors in *Kras*-mutated mouse models relative to mice carrying the wild-type *Kras* gene.^28^ This result confirmed the validity of expression profiling in our mouse models and demonstrated its potential utility for the analysis of downstream targets of the *KRAS* mutation in CRC.

In the current study, we aimed to identify new molecular targets for the development of therapeutic agents against *KRAS*-mutated CRC, using microarray data to compare gene expression between mouse models with and without colonic epithelium-specific *Kras* mutation. We identified a novel gene, regulator of calcineurin 2 (*RCAN2*), that was found to be downregulated in the tumors in *Kras*-mutated mouse models, which may inhibit calcineurin enzyme activity in CRC and exhibit tumor suppressor function. Here we report that *KRAS* mutation may promote cancer cell proliferation by decreasing the expression of *RCAN2*, and that calcineurin and NFAT signaling may serve as potential therapeutic targets for *KRAS*-mutated CRC.

## Results

### Identification of genes with altered expression in response to mutation by microarray analysis

**Figure 1:**
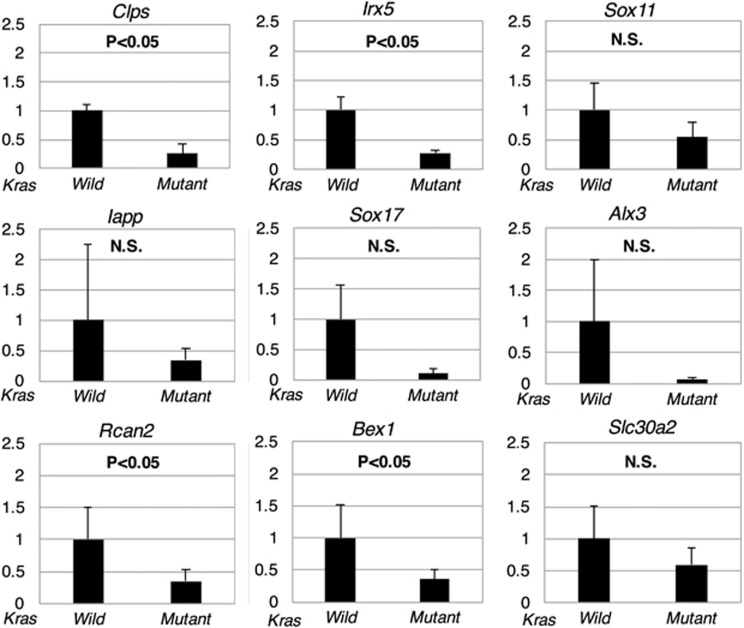
Comparison of gene expression in tumors from *Kras* wild-type and *Kras*-mutated mouse models using quantitative reverse transcription–PCR (qRT–PCR). Relative expression of *Rcan2* normalized to *B2m* was compared between *Kras* wild-type and *Kras*-mutated mouse models using qRT–PCR. Left bar; *CDX2P9.5-G22Cre;Apc*^*flox/flox*^*;LSL-Kras*^*G12D*^ mice (*n*=3), Right bar; *CDX2P9.5-G22Cre;Apc*^*flox/flox*^*;LSL-Kras*^*G12D*^ mice (*n*=3). Data are expressed as means+s.d. (error bars) of triplicate experiments showing relative expression of *Rcan2* normalized to that of *B2m*.

Genes that showed differential expression in the microarray analysis were identified by analysis of fold change and *P*-values. As genes with increased expression were previously reported by Kawaguchi *et al.*,^28^ we focused on downregulated genes. We analyzed 34 000 transcripts and found that the expression of 19 genes was significantly downregulated in tumors from *Kras*-mutated mice compared with those from *Kras* wild-type mice (Table 1). We set the fold change filter to 5.0 and the *P*-value at <0.05. Quantitative reverse transcription–PCR primers were designed for 9 of the 19 genes. The expression of four genes, namely *Clps*, *Irx5*, *Bex1* and *Rcan2,* was confirmed to be decreased in the *Kras*-mutated mouse group compared with the wild-type group (Figure 1).

### *KRAS* mutations are significantly associated with RCAN2 expression in human CRC

**Figure 2:**
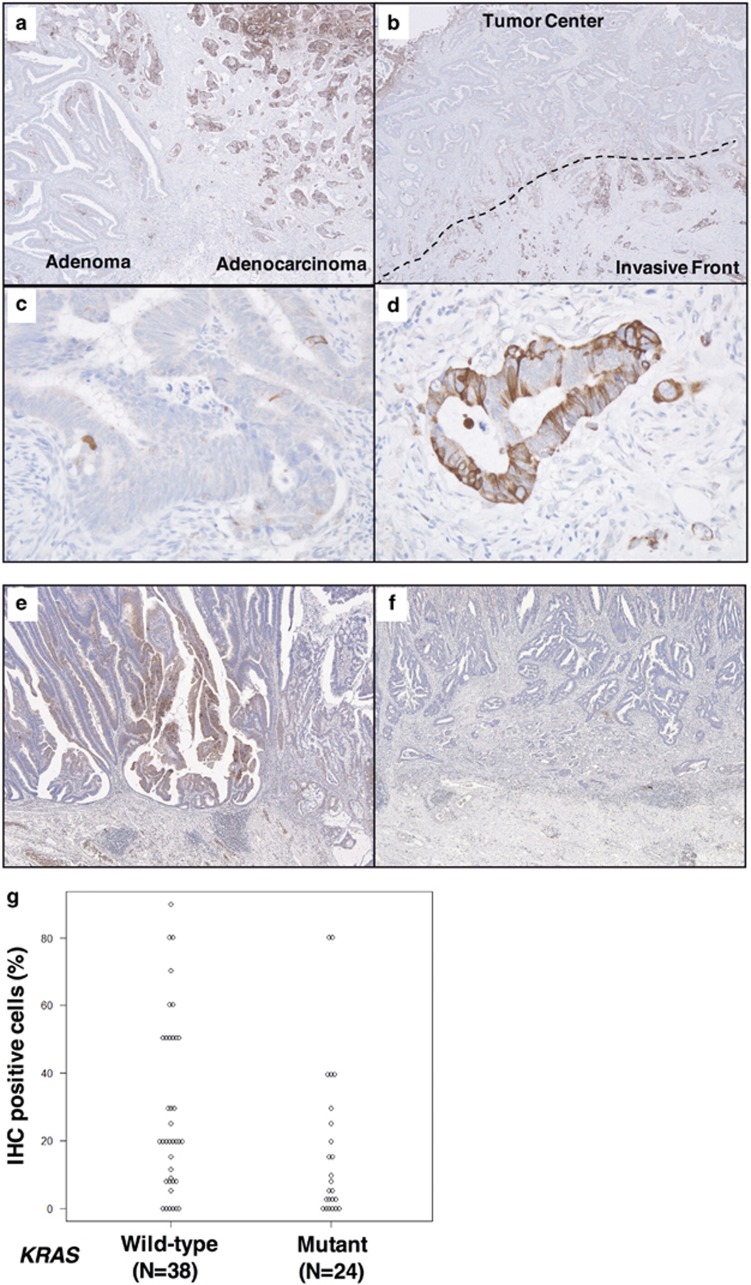
Immunohistochemical analyses of *RCAN2* in surgical specimens from patients with colorectal cancer (CRC). Representative images of immunohistochemical staining (IHC) at the border between adenoma (right, in **a**) and adenocarcinoma (left, in **a**), and the intratumoral distribution of *RCAN2* expression (**b**). Magnified views at the tumor center and the invasive front of tumors (**c**, **d**, respectively). Representative IHC images of *KRAS* wild-type and *KRAS-*mutated human CRC tissue samples (**e**, **f**, respectively). Comparison of IHC-positive cells in the carcinomatous area of *KRAS* wild-type and *KRAS-*mutated CRC indicated lower expression of *RCAN2* in *KRAS*-mutated CRC (*P*=0.0447, **g**).

Immunohistochemical staining (IHC) analysis of human CRC specimens revealed that RCAN2 was specifically expressed only in cancer cells but not in normal colonic epithelia, adenomas or tumor stroma (Figure 2a). Moreover, RCAN2 showed higher expression at the invasive front (IF) of tumors (defined as tumor cells or clusters within 500 μm of the IF) than at the tumor centers (Figures 2b–d).

**Figure 3:**
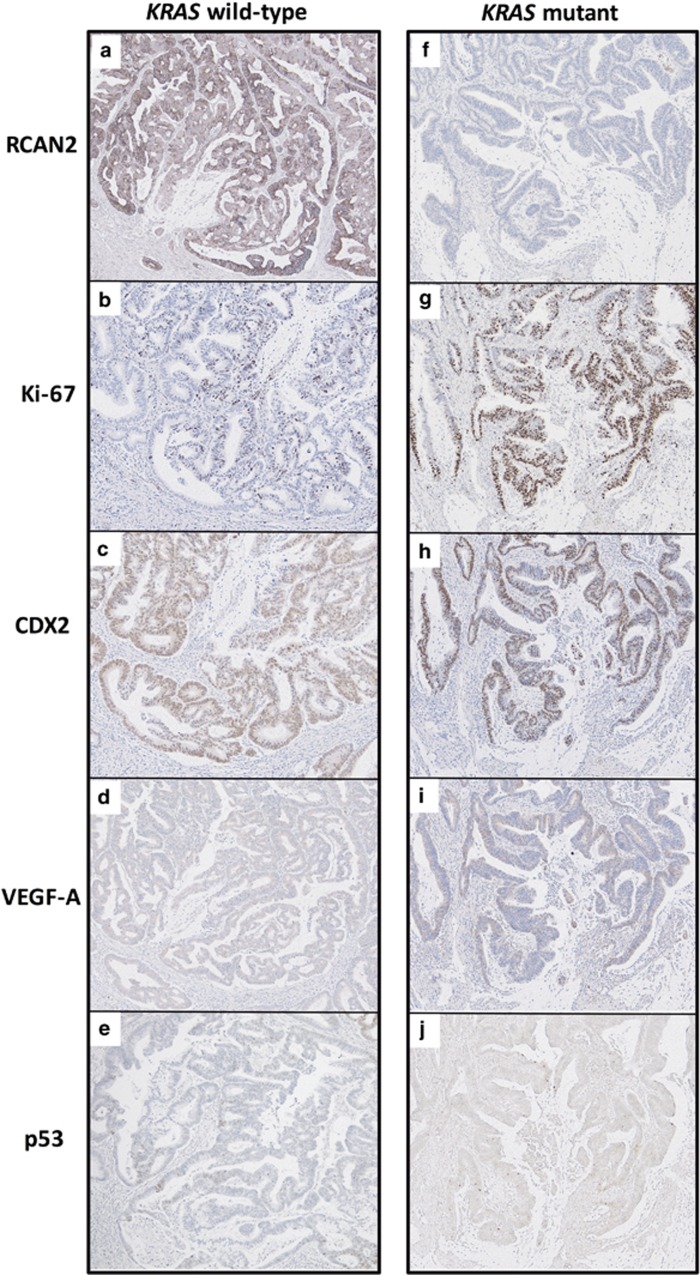
Immunohistochemical analyses of RCAN2 and other markers at invasive front of cancer. Representative IHC images of *KRAS* wild-type (**a**–**e**) and *KRAS-*mutated (**f**–**j**) human CRC tissue samples, stained with anti-RCAN2 (**a**, **f**), anti-Ki-67 (**b**, **g**), anti-CDX2 (**c**, **h**), anti-VEGF-A (**d**, **i**), and anti-p53 (**e**, **j**) antibodies.

Subsequently, we performed IHC to investigate the correlation between *KRAS* mutation and RCAN2 expression at the IF in early-stage human CRC specimens, as it is known that *KRAS* mutations occur relatively early during multistep carcinogenesis and that RCAN2 is potentially activated by various unknown stimuli. A total of 62 CRC specimens that did not involve the muscularis propria were genotyped by direct sequencing. *KRAS* mutations were identified in specimens from 24 patients (38.7%), and the remaining 38 specimens carried the wild-type *KRAS* gene (61.3%). Upon further analysis, a lower frequency of IHC-positive cells and a lower IHC-positive rate (cutoff >20%) were observed in specimens from patients with *KRAS*-mutated tumors (corresponding *P*-values 0.0447 and 0.362, respectively; Figures 2e–g and Table 2), whereas other clinicopathological characteristics were comparable between positive and negative expression of RCAN2 (Table 2). Furthermore, the correlation between RCAN2 expression at the IF of the tumor and other markers, including Ki-67, p53, CDX2 and VEGF-A, was studied by IHC. Ki-67 was upregulated with decreased expression of RCAN2 in the KRAS-mutated CRC, and there was no association between RCAN2 expression and the expression of other markers, except Ki-67 (representative image is shown in Figure 3).

### Effect of overexpression on cancer cell proliferation and migration

**Figure 4:**
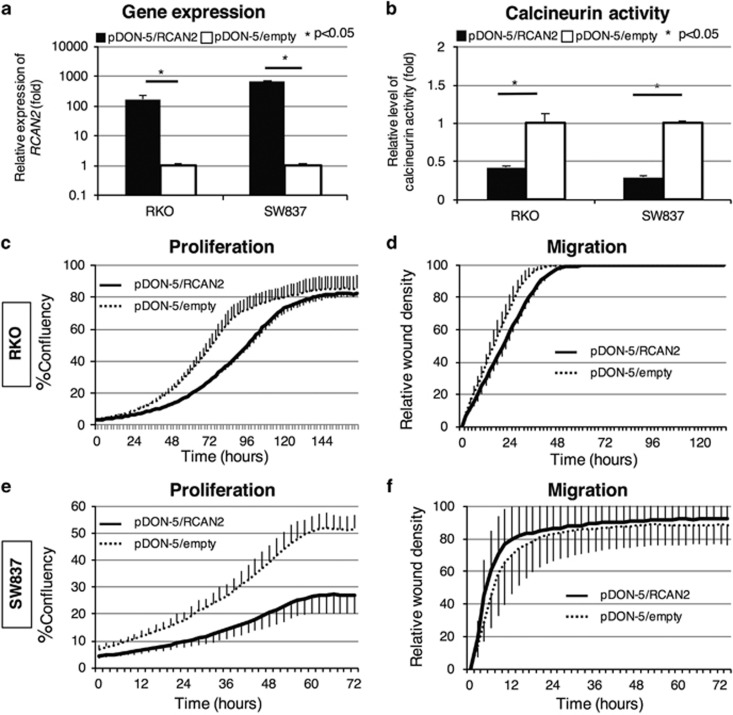
Overexpression of *RCAN2* in RKO and SW837 human colorectal cancer cell lines. RKO (*BRAF*-mutated) and SW837 (*KRAS*-mutated) cells showing weak expression of *RCAN2* were infected with RCAN2/pDON-5 and pDON-5/empty retroviruses. *RCAN2* expression (**a**) and calcineurin activity (**b**) were compared between cells infected with RCAN2/pDON-5 and pDON-5/empty (black and white bars, respectively). Proliferation and migration were compared in RKO cells with or without *RCAN2* overexpression (**c**, **d**, respectively) and SW837 cells with or without *RCAN2* overexpression (**e**, **f**, respectively).

We analyzed the effect of *RCAN2* expression on cancer cell proliferation and migration by overexpressing *RCAN2* in CRC cell lines. RKO (*BRAF*-mutated) and SW837 (*KRAS*-mutated) cells with low endogenous *RCAN2* mRNA levels (Supplementary Information 1) were infected with a retrovirus containing the pDON-5/RCAN2 vector for overexpression of *RCAN2* or an empty vector (pDON-5) as a control. Quantitative reverse transcription–PCR revealed that *RCAN2* was overexpressed in both cell lines infected with the pDON-5/RCAN2 vector (Figure 4a). Moreover, we observed decreased phosphatase activity of calcineurin in RKO and SW837 cell lines in which *RCAN2* was overexpressed (Figure 4b). The proliferation and wound-scratch migration assays performed using the IncuCyte Zoom system (Essen BioScience, Ann Arbor, MI, USA) revealed that cell proliferation was significantly suppressed in the pDON-5/RCAN2 group compared with the pDON-5 groups in both cell lines; however, no significant differences in migration activity were observed (Figures 4c–f).

### Effect of *RCAN2* shRNA knockdown on cancer cell proliferation and migration

**Figure 5:**
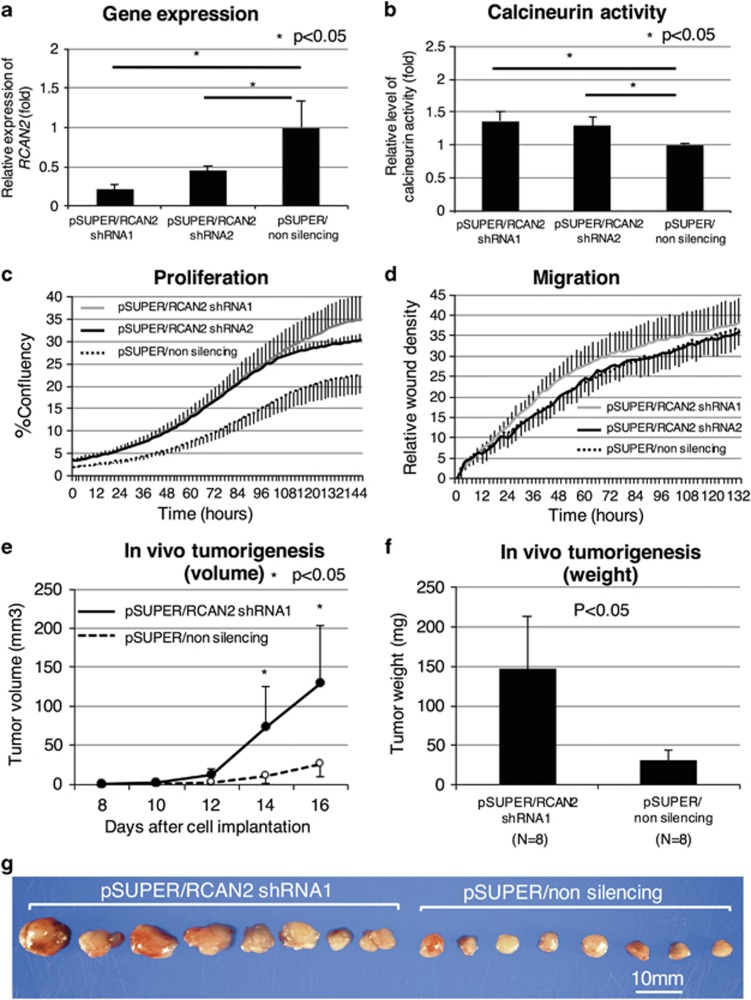
shRNA knockdown of *RCAN2* in the *Colo320* human colorectal cancer cell line. *RCAN2* expression in Colo320 cells (both *KRAS* and wild-type *BRAF*) with high expression of *RCAN2* was knocked down using an shRNA vector. *RCAN2* expression (**a**) and calcineurin activity (**b**) were compared between cells infected with pSUPER/RCAN2 1, 2 and pSUPER/non-silencing. These cells were subjected to proliferation and wound-scratch migration assays (**c**, **d**, respectively). *In vivo* tumorigenesis was compared between Colo320 cells infected with pSUPER/RCAN2 1 and pSUPER/non-silencing using a xenograft subcutaneous transplantation model in order to determine tumor volume (**e**) and tumor weight (**f**). Images of harvested tumors are shown in **g**.

Colo320 cells (both *KRAS* and *BRAF* wild-type) with relatively high endogenous *RCAN2* mRNA levels (Supplementary Information 1) were infected with retroviruses containing two short hairpin RNA (shRNA) vectors (pSUPER/RCAN2 shRNA1 and 2) or a non-silencing (pSUPER*/*non-silencing shRNA) vector. In cell lines infected with pSUPER/RCAN2, efficient knockdown of *RCAN2* was confirmed (Figure 5a). We also confirmed increased phosphatase activity of calcineurin in Colo320 cell lines infected with pSUPER/RCAN2 (Figure 5b). The proliferation and wound-scratch assays revealed that proliferation was significantly enhanced in both *RCAN2* shRNA groups compared with the non-silencing groups; however, no significant differences in migration activity were observed (Figures 5c and d). These results suggest that *RCAN2* represses the proliferation of cancer cells, but does not affect migration activity.

### *RCAN2* knockdown promotes xenograft tumor growth

In order to further investigate the impact of *RCAN2* on tumorigenesis, we implanted Colo320 cells infected with pSUPER/RCNA2 shRNA1 and pSUPER/non-silencing shRNA into nude mice (*n*=8 for each group). Tumor growth was observed significantly earlier in the pSUPER/RCAN2 group than in the pSUPER/non-silencing group (Figure 5e). Tumor weights were significantly higher in the pSUPER/RCAN2 group than in the pSUPER/non-silencing group (Figures 5f and g; pSUPER/RCAN2; 147 mg vs pSUPER/non-silencing; 31 mg, *P*=0.013). These results indicate that *RCAN2* silencing plays an important role in promoting cell proliferation during colorectal tumorigenesis *in vivo*.

### RCAN2 function is dependent on the calcineurin pathway

**Figure 6:**
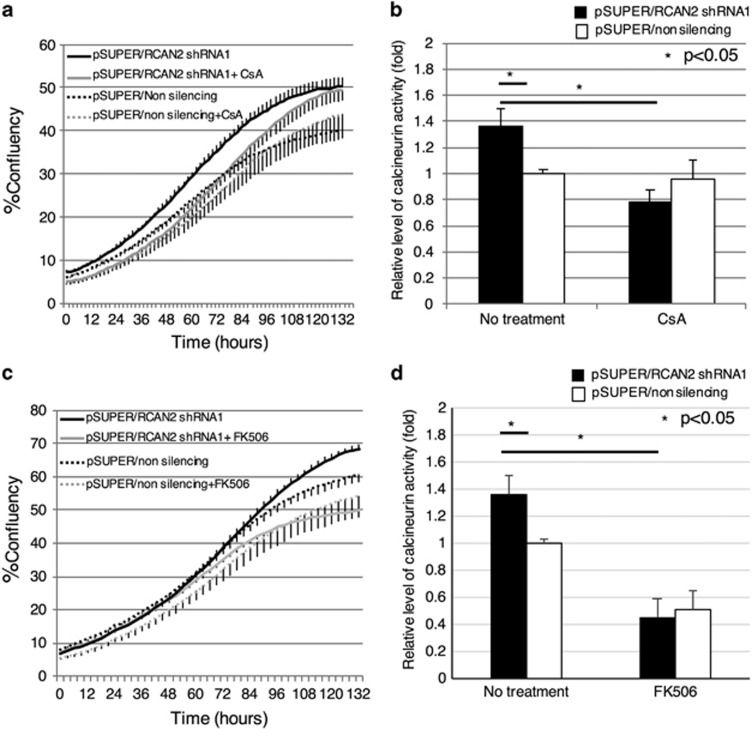
Proliferation assay in Colo320 cells, with or without *RCAN2* shRNA knockdown, following calcineurin inhibitor treatment. Colo320 cells infected with pSUPER/RCAN2 shRNA1 and pSUPER/non-silencing shRNA were cultured in medium with or without either cyclosporine A or tacrolimus, and cell proliferation (**a**, **c**, respectively) and enzyme activity (**b**, **d**, respectively) were subsequently analyzed. Black line: pSUPER/RCAN2 shRNA1 without treatment; gray line: pSUPER/RCAN2 shRNA1 with calcineurin inhibitors; black dashed line: pSUPER/non-silencing shRNA without treatment; black dashed line: pSUPER/non-silencing shRNA with calcineurin inhibitors. CsA, cyclosporine A; FK506, tacrolimus.

In order to confirm that the function of RCAN2 is dependent on the calcineurin–NFAT pathway, Colo320 cells infected with both pSUPER/RCAN2 shRNA1 and pSUPER/non-silencing shRNA were treated with a CNI, cyclosporine A (0.1 μm) or tacrolimus (0.5 μm). Proliferation in the pSUPER/RCAN2 group was repressed by treatment with cyclosporine A in accordance with decreased enzyme activity, whereas cyclosporine A did not have an effect in pSUPER/non-silencing (Figures 6a and b). For tacrolimus, the difference in proliferation between pSUPER/RCAN2 and pSUPER/non-silence groups disappeared after treatment with tacrolimus. Moreover, although enzyme activity was decreased in both groups to a lower level than that in the pSUPER/non-silencing group without any treatment, proliferation was repressed in an enzyme activity-dependent manner (Figures 6c and d). These results suggest that the function of *RCAN2* is dependent on the calcineurin–NFAT pathway.

## Discussion

In this study, following microarray analysis of tissue samples from the two mouse models and subsequent validation by quantitative reverse transcription–PCR, four candidate genes were identified as potential downstream targets of oncogenic mutated *KRAS*. Of these four candidates, we focused on *RCAN2*, an endogenous calcineurin regulator, as increased expression of calcineurin has been reported in human CRC by IHC analyses and comprehensive protein expression profiling using antibody microarrays.^23, 24^ Moreover, cyclosporine A, which is a known specific inhibitor of calcineurin, has been reported to inhibit the proliferation of CRC cell lines (HT29 and Wider) by regulating *c-Myc, p21(WAF1/CIP1)*, and proliferating cell nuclear antigen in a dose-dependent manner.^30^ Therefore, the calcineurin–NFAT pathway was hypothesized to have an important role in human CRC. To date, the association between oncogenic mutated *KRAS* and calcineurin signaling has not been described. To our knowledge, the current study is the first report that oncogenic mutated *KRAS* and calcineurin signaling are associated via changes in the expression of *RCAN2.*

*RCAN2* was first cloned as a thyroid hormone-responsive gene, *ZAKI-4,* from human fibroblasts.^31^ This family of genes includes *ZAKI-4,* Down syndrome critical region 1 *(DSCR1)*,^32^ and Down syndrome critical region 1 like-2 *(DSCR1L2)*,^33^ which share 61–68% identity in amino acid sequence.^33^ These genes have been demonstrated to inhibit calcineurin activity by binding the catalytic subunit of calcineurin.^33, 34, 35, 36^ This gene family, whose members are collectively known as regulators of calcineurin *(RCAN)*, comprises *RCAN1*, *RCAN2* and *RCAN3* (previously known as *DSCR1, ZAKI-4* and *DSCR1L2*, respectively*). RCAN2* has three transcriptional variants and two protein isoforms; *RCAN2* transcript variant α encodes isoform-α and transcript variants β1 and β2 encode isoform-β. Both isoforms have identical C-terminals and different N-terminals. The α-transcripts are present only in the brain, whereas β-transcripts are ubiquitous but most abundant in the brain, heart, skeletal muscle and kidneys. The expression of α-transcripts, but not that of β-transcripts, is promoted by the thyroid hormone.^37, 38^ In the present study, quantitative reverse transcription–PCR using primers specific for both the α- and β-transcripts of *RCAN2* was performed (data not shown), but only β-transcripts were observed to be expressed in mouse colonic epithelium and tumors.

The functions of genes in the *RCAN* family have been studied in endothelial cells. Except for the *RCAN1-1L* isoform, which promotes angiogenesis, the overexpression of *RCAN1-4*, *RCAN2* and *RCAN3* in human umbilical vein endothelial cells has been reported to inhibit cell proliferation and migration.^39, 40, 41^ A correlation between *RCAN2* and bone turnover,^42^ obesity^43^ and response to anabolic steroid administration^44^ has also been reported in organs in which its expression is abundant; however, to date, the correlation between *RCAN2* and cancer has not been studied. Moreover, the contribution of other members of the *RCAN* family to cancer development is not well-known. A few studies have reported that *RCAN1* is expressed in tumor vessels, but not in tumor cells, in renal and ovarian cancers.^39, 45^ Our finding that *RCAN2* is specifically expressed in human CRC and has an inhibitory role in cancer cell proliferation is therefore novel.

IHC staining revealed that *RCAN2* was expressed mainly at the tumor IF in tumor cells and that the expression of this gene was repressed by oncogenic mutation of *KRAS* in human CRC. However, the reasons for the predominance of *RCAN2* expression at the IF remain unclear. One possibility is that *RCAN2* may predominantly be expressed in highly proliferative cells, which are located at the IF, as part of a negative feedback loop. However, Ki-67, known as a marker of cell proliferation, was repressed simultaneously with RCAN2 expression, indicating that this hypothesis was incorrect. Another possibility is that interstitial interactions may affect expression. Gene expression profile comparisons between *KRAS* wild-type and *KRAS*-mutated CRC in cultured cell lines revealed that *RCAN2* expression does not decrease in *KRAS*-mutated CRC. However, a decrease in the expression of this gene was observed in our mouse models, those are thought to be conserved in terms of the cancer microenvironment. This finding supports the hypothesis that interstitial interactions may influence *RCAN2* expression. Moreover, *RCAN2* was predominantly expressed at the IF, even in mucosal adenocarcinoma or adenocarcinoma with slight submucosal invasion, where cancer cells and the interstitium share a margin. Although further investigation of the correlation between expression in cancer cells and in the interstitium is required, our results suggested that *RCAN2* is predominantly expressed at the IF and that *KRAS*-mutated CRC may promote tumor development due to decreased expression of *RCAN2*.

The present study has some limitations. First, the molecular mechanism by which mutated *KRAS* decreases the expression of *RCAN2* remains unclear. Although *KRAS*^*G12V*^ mutation and *KRAS*^*G12V*^ mutation with effector domain mutation^46^ were induced in CRC cell lines (such as Colo320 and Caco-2) containing wild-type *KRAS* and *BRAF* alleles, these mutations did not lead to decreased expression of *RCAN2* (data not shown). The microenvironment may affect *RCAN2* expression, and further studies are required in which *RCAN2* expression is compared in mouse models with colon epithelial cell-specific inactivation of *APC* and activation of other molecules downstream of *KRAS,* including *BRAF*, *PI3K* and *RALGDS*. Second, although the results of our functional studies of *RCAN2* suggest that *RCAN2* expression represses cell proliferation by inhibiting calcineurin phosphatase activity, a previous report suggested that cyclosporine A inhibits the growth of a colon cancer cell line independent of the calcineurin pathway and that tacrolimus promotes tumor growth despite efficient inhibition of NFAT activity.^47^ In the present study, we utilized CNIs to confirm that the tumor suppressor function of *RCAN2* is dependent on the calcineurin–NFAT pathway. For cyclosporine A, growth inhibition was observed only in Colo320 cell lines with *RCAN2* shRNA knockdown, and not in Colo320 cell lines without *RCAN2* knockdown. For tacrolimus, although both enzyme activity and proliferation were repressed by the treatment, proliferation was repressed in an enzyme activity-dependent manner. Therefore, we consider that our results support the tumor suppressor function of *RCAN2*. However, our findings represent only one of the various potential mechanisms for tumor growth in *KRAS*-mutated CRC. For instance, when considering CNIs as a targeted therapeutic agent for *KRAS*-mutated CRC, the impact of immunosuppression on tumor development should be considered. However, some agents such as cyclophosphamide, methotrexate and everolimus are known as immunosuppressant and chemotherapeutic drugs. Dosage might be important because these agents act as immunosuppressants at lower doses, whereas they act as chemotherapeutic drugs at higher doses. *In vivo* experiments using mouse models are expected to be useful to address this issue. We have already developed a genome-engineered mouse model that sporadically generates colon cancers with the *Kras* mutation. This model could be used to evaluate the effect of CNI on both CRC and the immune system when administering CNIs in various dosages.

In conclusion, *KRAS* mutation in CRC leads to decreased expression of *RCAN2*, resulting in tumor proliferation by derepression of the calcineurin–NFAT signaling pathway. The RCAN2–calcineurin–NFAT pathway may potentially serve as a novel molecular target for the development of therapeutic agents against *KRAS*-mutated CRC and should be further investigated using the previously described mouse models of sporadic colon cancer.

## Materials and methods

### Animal experiments

All animal experiments were approved by the University Committee on the Use and Care of Animals of Hiroshima University and performed according to Japanese regulations based on the 1964 Declaration of Helsinki Principles and its later amendments. All mice were housed under specific pathogen-free conditions. Four or five mice were housed per cage with chopped wood bedding, and sufficient food and water (with 10.0 mg/l of chlorine) were supplied in each cage. The breeding room was maintained at a constant temperature of 23±2 °C, relative humidity of 50±5%, with 15–20 air changes per hour, and a 12-h light/dark cycle with lights on at 0800 hours. Necropsy and tumor removal for tumorigenesis experiments were performed after killing with intraperitoneal injection of pentobarbital followed by cervical dislocation.

### Gene expression profiling

We used microarray data previously obtained by Kawaguchi *et al.*^28^ from a comparison between the two mouse models *CDX2P9.5-G22Cre;Apc*^*flox/flox*^*;LSL-Kras*^*G12D*^ (C57BL/6J) and *CDX2P9.5-G22Cre;Apc*^*flox/flox*^*;Kras* wild-type (C57BL/6J). Briefly, the 9.5-kb promoter sequence of *CDX2* exhibits transcriptional activity specifically in the mouse colonic epithelium. Therefore, we used the transgene *CDX2P9.5-G22Cre* for colonic epithelium-specific inactivation of *Apc* and activation of *Kras* via the loxP/Cre system. In the mouse models, a 22-guanine nucleotide tract (G22) that alters the reading frame was inserted and activated stochastically in the mouse intestinal tract, producing a microsatellite-unstable phenotype. Colon cancers were generated at the proximal colon and cecum, with minimal morphological differences between the two mouse models. At the age of 6–8 weeks, 3 tumors were collected from each of the 2 mouse models and gene expression profiles of 34 000 transcripts were generated using microarrays (GeneChip Mouse Genome 430 2.0 Array; Affymetrix, Santa Clara, CA, USA). The microarray data are available in the NCBI GEO database (Accession number: GSE75435)

### Quantitative reverse transcription–PCR

Complementary DNA was generated using a QuantiTect Reverse Transcription Kit (Qiagen, Hilden, Germany) and amplified using a Rotor-Gene Q 2PLEX HRM Real-Time PCR system (Qiagen). The quantitative PCR reactions, which were prepared to a final volume of 25 μl, included 2 × Rotor-Gene SYBR Green PCR Mix (Qiagen), 10 μmol/l forward/reverse primers, and 32 ng of each complementary DNA sample. The amplification protocol involved denaturation at 95 °C for 5 min, followed by 40 cycles at 95 °C for 5 s and 60 °C for 10 s. Beta-2-microglobulin (*B2M*) was used as an internal control. The primers used are shown in 'Supplementary Information 2'.

### Tissue samples

Formalin-fixed paraffin-embedded human CRC specimens, those were obtained from the patients who had undergone colectomy at Hiroshima University Hospital between 2006 and 2011, were used for *KRAS* genotyping an IHC analyses. Of these, specimens that did not involve the muscularis propria were included. Comprehensive approvals for basic or clinical research were obtained from all of the patients. Experiments using human subjects were performed in accordance with the Ethical Guidelines for Human Genome/Gene Research set forth by the Japanese Government, and the study was performed with the permission of the Ethics Committee of Hiroshima University.

### *KRAS* genotyping

For human CRC specimens, DNA was extracted from formalin-fixed, paraffin-embedded tumor tissue sections using TaKaRa DEXPAT (Takara Bio Inc., Shiga, Japan). Exon 2 of *KRAS* was amplified by PCR, and the products were directly sequenced using an ABI 3130 Genetic Analyzer (Applied Biosystems, Foster City, CA, USA) according to the manufacturer's instructions.

### IHC staining

A Dako LSAB Kit (Dako) was used for IHC analysis. Sections were pretreated by microwave treatment in citrate buffer for 15 min to retrieve antigenicity. After peroxidase activity was blocked with 3% H_2_O_2_–methanol for 10 min, sections were incubated with normal goat serum (Dako) for 20 min to block non-specific antibody-binding sites. Sections were incubated with the following primary antibodies: anti-RCAN2 (Thermo Scientific, Rockford, IL, USA; diluted 1:500), anti-Ki-67 (clone MIB-1; Dako, Carpinteria, CA, USA; diluted 1:50), anti-CDX2 (clone AMT28; BioGenex, San Ramon, CA, USA; diluted 1:20), anti-VEGF-A (Santa Cruz Biotechnologies, Santa Cruz, CA, USA; diluted 1:50), and anti-p53 (clone DO-7; Novocastra, Newcastle, UK; diluted 1:50). Sections were incubated with primary antibody for 1 h at 25 °C, followed by incubation with biotinylated anti-rabbit or anti-mouse immunoglobulin G and peroxidase-labeled streptavidin for 10 min each. Staining was completed with a 10-min incubation in substrate–chromogen solution. The sections were counterstained with 0.1% hematoxylin. Appropriate positive and negative control samples were also stained.

### Cell lines

All other cell lines were obtained from the American Type Culture Collection (ATCC) between 1998 and 2000. The amphotropic Phoenix packaging cell line was provided by G. Nolan (Stanford University, Stanford, CA, USA). Details of cell culture conditions were previously described.^48^

### Plasmid construction

In brief, a 732-bp fragment of the *RCAN2* coding sequence was amplified by PCR using complementary DNA from a normal colonic epithelium and then inserted into the retroviral vector pDON-5 neo (TaKaRa) to generate the pDON-5/RCAN2 vector for overexpression of *RCAN2*. Hairpin-loop oligonucleotides targeting *RCAN2* (Supplementary Information 2) and a non-silencing sequence were synthesized and inserted into pSUPER.retro.neo+gfp (OligoEngine, Seattle, CA, USA) to generate ‘pSUPER/RCAN2 *shRNA1, 2* and ‘pSUPER/non-silencing shRNA'. All plasmid products were verified by sequencing.

### Retroviral infection

The Phoenix packaging cells were transfected with the retroviral constructs; the supernatant containing nonreplicating amphotropic retroviruses was collected. For *RCAN2* overexpression, RKO (*BRAF*-mutated) and SW837 (*KRAS-*mutated) cell lines were infected with retroviruses containing pDON-5/RCAN2 and pDON-5 empty vectors. For *RCAN2* silencing, the Colo320 cell line (wild type for both *KRAS* and *BRAF*) was infected with retroviruses containing pSUPER/RCAN2 shRNA1, 2 and pSUPER/non-silencing shRNA vectors. Cells were selected using Geneticin (G418) at concentrations of 1000, 750 and 750 μg/ml for RKO, SW837 and Colo320 cells, respectively, for 2 weeks.

### Cellular proliferation and migration assay

Cell proliferation and wound-scratch migration were measured using a brightfield image label-free high-content time-lapse assay system (IncuCyte Zoom system; Essen BioScience) according to the manufacturer's instructions. In brief, for the proliferation assay, equal numbers of cells (1 × 10^5^/well) were seeded on to 24-well plates in the appropriate culture medium with supplements or agents, and percent cell confluence was then continuously measured using the IncuCyte system over a 5-day period. For the migration assay, cells (1.2 × 10^5^ cells/well) were seeded on to 96-well ImageLock tissue culture plates (Essen BioScience) pre-incubated with type I collagen. Once cells reached >90% confluence, at 12 h after seeding, wound scratches were made using a 96-pin WoundMaker (Essen BioScience) and relative wound densities were measured using the IncuCyte system over a 5-day period.

### *In vivo* tumorigenesis assay

Five-week-old female BALB/cA Jcl-nu mice (CLEA Japan, Tokyo, Japan) were used for the *in vivo* tumorigenesis assay. According to the guide line for the welfare and use of animals in cancer research,^49^ sample size for animal expreiments were determined as eight mice in each group. A total of 1.0 × 10^7^ Colo320 cells containing a retrovirus expressing *RCAN2* shRNA1 or non-silencing shRNA were subcutaneously injected into the right flanks of eight nude mice in each group. Neither randomization nor blinding for animal use were performed because we commercially obtained these mice with the same genetic background. Tumor size was measured using Vernier calipers every 2 days (from day 8 to day 16) after cell implantation. Tumor volume was calculated using the formula: V=0.5 a × b^2^, where a represents the long diameter and b the short diameter of the tumor. Tumors were removed from the mice and weighed on day 16.

### Calcineurin activity assay

Calcineurin activity was measured using a calcineurin cellular activity assay (Enzo Life Sciences, Farmingdale, NY, USA) according to the manufacturer's instructions. Extracted cell lysates were centrifuged at 100 000 *g* for 45 min at 4 °C and the supernatant was placed in a desalting column to remove excess phosphates and nucleotides. Total phosphatase activity was assessed in the samples by incubating with a phosphopeptide substrate. Calcium-independent phosphatase activity was assessed in the samples by incubating samples with EGTA and a phosphopeptide substrate. The resulting total phosphatase activity was subtracted from the calcium-independent phosphatase activity to determine calcineurin activity levels.

### Treatment of cells with CNIs

Cyclosporine A and tacrolimus were purchased from Novartis Pharma Japan and Astellas Pharma Japan, respectively. For the proliferation assay, cells were cultured in medium supplemented with 0.1 μm cyclosporine A or 0.5 μm tacrolimus until the end of the IncuCyte proliferation assay. To confirm the suppression of calcineurin activity under the treatment with cyclosporine A and tacrolimus, cell lysates were extracted 96 h after treatment with CNIs and calcineurin activity was compared between extracted cell lines with or without the treatment of CNIs.

### Statistical analysis

All experiments were repeated at least three times with each sample in triplicate. Sample sizes for relevant experiment were determined by power analysis. All values are expressed as means±s.d. The statistical significance of differences was determined using the Student's *t*-test, Mann–Whitney *U*-test, or Fisher's exact test. A two-tailed *P*-value of <0.05 was considered statistically significant. All statistical analyses were performed using JMP 10 software (SAS Institute Inc., Cary, NC, USA) and R statistical software version R2.10.0 (R Foundation for Statistical Computing, Vienna, Austria).

## Figures and Tables

**Table 1:** Gene expression profiling using microarray analysis for *Kras* wild-type and *Kras*-mutated mouse models (downregulated genes)

*Gene symbol*	*Gene title*	*Fold change*	*Function*
*S100g*	S100 calcium binding protein G	57.23	Calcium-binding protein
*Clps*	Colipase, pancreatic	19.96	Co-enzyme
*Irx5*	Iroquois related homeobox 5 (*Drosophila*)	11.49	Homeobox gene
*Defa5*	Defensin, alpha, 5	10.55	Antimicrobial peptide
*Svopl*	SV2-related protein homolog (rat)-like	10.43	Transmembrane transporter
*Sox11*	SRY box-containing gene 11	9.83	Transcription factor
*Iapp*	Islet amyloid polypeptide	9.63	Peptide hormone
*Sox17*	SRY box-containing gene 17	9.50	Transcription factor
*Alx3*	Aristaless-like homeobox 3	8.58	Homeobox gene
*Gbx2*	Gastrulation brain homeobox 2	7.97	Homeobox gene
*Shh*	Sonic hedgehog	7.91	Growth factor
*H19*	H19 fetal liver mRNA	7.79	Long noncoding RNA, regulating cell proliferation
*Ceacam10*	Carcinoembryonic antigen-related cell adhesion molecule 10	7.11	Cell adhesion molecule
*Rcan2*	Regulator of calcineurin 2	6.28	Signaling molecule regulating calcineurin activity
*Bex1*	Brain expressed gene 1	6.23	Signaling adapter molecule
*Asprv1*	Aspartic peptidase, retroviral-like 1	5.93	Aspartic protease
*Nrcam*	Neuron-glia-CAM-related cell adhesion molecule	5.92	Cell adhesion molecule
*Wnt10a*	Wingless related MMTV integration site 10a	5.72	Growth factor
*Slc30a2*	Solute carrier family 30 (zinc transporter), member 2	5.09	Zinc transporter

**Table 2:** Comparison of clinicopathological characteristics between positive and negative *RCAN2* expression in human colorectal cancer

	*RCAN2 (positive: >20%)*		P*-value*
	*Positive (*N=*32)*	*Negative (*N=*30)*	
*KRAS status (n)*			
Wild-type	24	14	0.0362
Mutant	8	16	

*Age(years)*			
Median (range)	71 (45–86)	68 (40–96)	0.174

*Sex*			
Male	17	15	1
Female	15	15	

*Tumor location*			
Right sided	23	24	0.558
Left sided	9	68 (40–96)	

*Histologic type*			
Papillary	5	5	0.989
Well differentiated	18	17	
Moderate differentiated	9	8	

*Vascular invasion*			
Absent	27	27	0.709
Present	5	3	

*Lymphatic invasion*			
Absent	26	26	0.733
Present	6	4	

*Nodal metastasis*			
Absent	30	27	0.733
Present	2	3	

Abbreviations:*KRAS*, Kirsten rat sarcoma viral oncogene homolog; *RCAN2*, regulator of calcineurin 2.
