# Supplementary material for: KRAS mutation leads to decreased expression of regulator of calcineurin 2, resulting in tumor proliferation in colorectal cancer
Source: Oncogenesis. 2016 Aug 15;5(8):e253–. doi: 10.1038/oncsis.2016.47 (PMC5007825; doi:10.1038/oncsis.2016.47)
Supplement: Supplementary Information legends [file oncsis201647x1.docx]

Supplementary Information 2

1. Primers fro qRT-PCR(mouse)

| ***Primer*** | ***Sequence(5'-3')*** |
| --- | --- |
| Clps_Forward | aacttccagcttccatccac |
| Clps_Reverse | caaacagatctcaccgtcctc |
|  |  |
| Irx5_Forward | aaccagacggtgttgaatcg |
| Irx5_Reverse | aactcatagggggagtctttgc |
|  |  |
| Sox11_Forward | cggtgatgaaatgttgttgg |
| Sox11_Reverse | acacgataaaggacgggaag |
|  |  |
| Iapp_Forward | acttgggctgtagttcctgaag |
| Iapp_Reverse | ctcaagatccctttcagtggtc |
|  |  |
| Sox17_Forward | tgaacgcctttatggtgtgg |
| Sox17_Reverse | cttctctgccaaggtcaacg |
|  |  |
| Alx3_Forward | ctggctttgcgaacagacct |
| Alx3_Reverse | tgggcagtacggagatgtca |
|  |  |
| Rcan2_Foward | ctccccaactcattgtttgc |
| Rcan2_Reverse | ttcatcataggtccggaacag |
|  |  |
| Bex1_Forward | aagaggagaaggcaaggatagg |
| Bex1_Reverse | gctcccttctgatggtatcttg |
|  |  |
| Slc30a2_Forward | agccctgcatagcctgcata |
| Slc30a2_Reverse | agcattctgggcaatggcta |

1. Oligonucleotides targeting *RCAN2* for shRNA

| ***Primer*** | ***Sequence(5'-3')*** |
| --- | --- |
|  |  |
| RCAN2-shRNA1_Forward | gatccccgtgcattacagaagatacattcaagagatgtatcttctgtaatgcacttttta |
| RCAN2-shRNA1_Reverse | agcttaaaaagtgcattacagaagatacatctcttgaatgtatcttctgtaatgcacggg |
|  |  |
| RCAN2-shRNA2_Forward | gatccccgagcttagacttcaaatatttcaagagaatatttgaagtctaagctcttttta |
| RCAN2-shRNA2_Reverse | agcttaaaaagagcttagacttcaaatattctcttgaaatatttgaagtctaagctcggg |
