# Supplementary figures and images for: KRAS mutation leads to decreased expression of regulator of calcineurin 2, resulting in tumor proliferation in colorectal cancer
Source: Oncogenesis. 2016 Aug 15;5(8):e253–. doi: 10.1038/oncsis.2016.47 (PMC5007825; doi:10.1038/oncsis.2016.47)

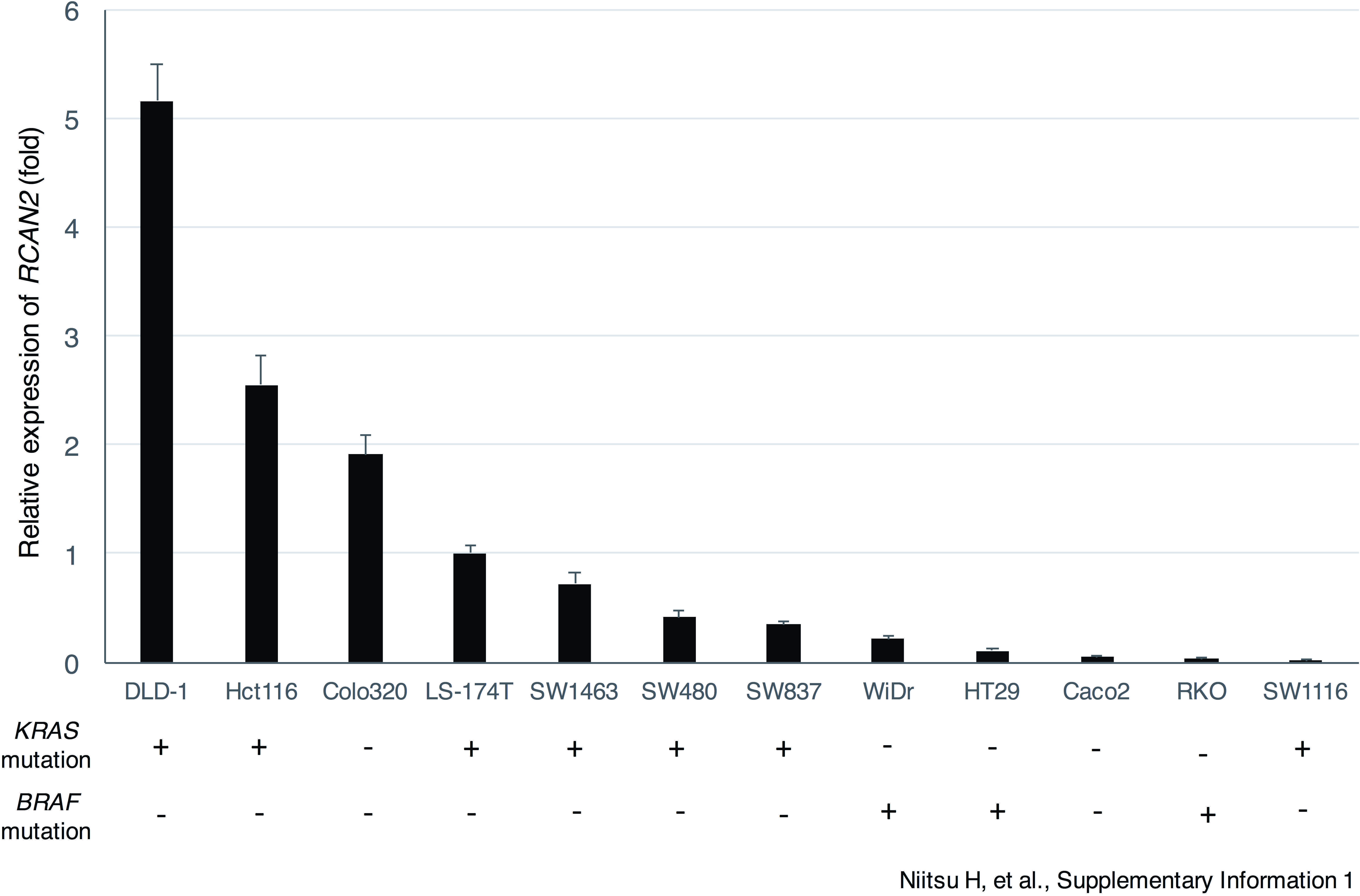

Supplement: Supplementary Information 1 [file oncsis201647x2.tif]
