# Supplementary material for: KRAS mutation leads to decreased expression of regulator of calcineurin 2, resulting in tumor proliferation in colorectal cancer
Source: Oncogenesis. 2016 Aug 15;5(8):e253–. doi: 10.1038/oncsis.2016.47 (PMC5007825; doi:10.1038/oncsis.2016.47)
Supplement: Supplementary Information 2 [file oncsis201647x3.docx]

**Supplementary Information legends**

Supplementary Information 1. Relative expression of *RCAN2* normalized to *B2M* expression in human colorectal cancer cell lines.

White bar: both *KRAS* and *BRAF* wild-type, Black bar: *KRAS-*mutated, Shaded bar: *BRAF*-mutated. Data are expressed as means + SD (error bars) of triplicate experiments.

Supplementary Information 2. Oligonucleotide sequences

A: primers for qRT-PCR, B: oligonucleotide targeting *RCAN2* for shRNA
